# Neural stem cell self-renewal stimulation by store-operated calcium entries in adult mouse area postrema: influence of leptin

**DOI:** 10.3389/fncel.2023.1200360

**Published:** 2023-06-09

**Authors:** Cyrine Ben Dhaou, Elodie Terrié, Nadine Déliot, Thomas Harnois, Laetitia Cousin, Patricia Arnault, Bruno Constantin, Emmanuel Moyse, Valérie Coronas

**Affiliations:** ^1^University of Tours, INRAe Centre Val-de-Loire UMR-85, CNRS UMR-1247, Physiologie de la Reproduction et Comportements, Nouzilly, France; ^2^4CS, Laboratory Channels and Connexins in Cancers and Cell Stemness, CNRS UMR 6041, University of Poitiers, Poitiers, France

**Keywords:** calcium channel, store-operated channel, leptin, circumventricular organ, brain, adult, neural stem cell

## Abstract

Neural stem cells (NSCs) persist in specific brain germinative niches and sustain neurogenesis throughout life in adult mammals. In addition to the two major stem cell niches in the subventricular zone and the hippocampal dentate gyrus, the area postrema located in the brainstem has been identified as a neurogenic zone as well. NSCs are regulated by signals from the microenvironment that adjust stem cell response to the needs of the organism. Evidence accumulated over the past decade indicates that Ca^2+^ channels play pivotal functions in NSC maintenance. In this study, we explored in area postrema NSCs the presence and roles of a subset of Ca^2+^ channels, the store-operated Ca^2+^ channels (SOCs) that have the capacity to transduce extracellular signals into Ca^2+^ signals. Our data show that NSCs derived from the area postrema express TRPC1 and Orai1, known to form SOCs, as well as their activator STIM1. Ca^2+^ imaging indicated that NSCs exhibit store-operated Ca^2+^ entries (SOCEs). Pharmacological blockade of SOCEs with SKF-96365, YM-58483 (also known as BTP2) or GSK-7975A resulted in decreased NSC proliferation and self-renewal, indicating a major role for SOCs in maintaining NSC activity within the area postrema. Furthermore, our results show that leptin, an adipose tissue-derived hormone whose ability to control energy homeostasis is dependent on the area postrema, decreased SOCEs and reduced self-renewal of NSCs in the area postrema. As aberrant SOC function has been linked to an increasing number of diseases, including brain disorders, our study opens new perspectives for NSCs in brain pathophysiology.

## 1. Introduction

Neural stem cells (NSCs) reside in defined niches in the adult brain where they produce new neurons and glial cells throughout life (Kumar et al., 2019). In addition to the two major stem cell niches in the subventricular zone and the dentate gyrus, the area postrema, a circumventricular organ located in the brainstem, has recently been identified as a neurogenic zone containing NSCs that serve as a source for producing new neurons and glial cells under homeostatic conditions (Bauer et al., 2005; Bennett et al., 2009; Furube et al., 2015). Like NSCs in major neurogenic niches, stem cells in the area postrema express the classical NSC markers, namely SOX2, nestin, the class III intermediate filament protein vimentin as well as GFAP (Pecchi et al., 2007). *In vitro*, area postrema NSCs have the capacity to proliferate and give rise to neurons and glial cells (Charrier et al., 2006; Bennett et al., 2009). *In vivo*, they produce new neurons and glial cells under homeostatic physiological conditions (Bauer et al., 2005) and are recruited by brain lesions (Sanin et al., 2013), as expected for a stem cell residing in the brain. Of note, a fivefold increase in the number of proliferating cells expressing Ki67 in the area postrema was observed in the brains of stroke patients, suggesting that NSCs in the area postrema may contribute to neurogenesis and plasticity in the human brain after injury (Sanin et al., 2013). As a major center of energy homeostasis control, the area postrema is a target of leptin, a long-term regulator of energy balance (Hayes et al., 2010), and amylin, a satiety hormone which promotes neurogenesis (Liberini et al., 2016a) while stress decreases neurogenic proliferation and NSC frequency (Chigr et al., 2009).

Ca^2+^, as an intracellular second messenger, controls a remarkable diversity of biological processes occurring throughout life, including genesis and homeostasis of organs and tissues (Berridge et al., 2003). One of the primary sources of Ca^2+^ signals in a wide variety of cells, particularly in non-excitable cells, is Ca^2+^ entry through store-operated Ca^2+^ channels (SOCs). SOCs are so named because they are activated by the emptying of intracellular Ca^2+^ stores of the endoplasmic reticulum (ER) (Parekh and Putney, 2005; Lopez et al., 2020; Emrich et al., 2022). SOCs are located in the plasma membrane and allow extracellular Ca^2+^ entry. These Ca^2+^ channels of the plasma membrane are mainly formed of Orai1 and are activated by STIM1 proteins, which are located in the ER membrane and act as a Ca^2+^ sensor. This process may also recruit cationic channels formed of four TRPC1 proteins, that could also act as SOCs. Ca^2+^ entry through SOCs is triggered after stimulation of cell surface receptors that act through G proteins or a tyrosine kinase cascade to activate phospholipase C and produce inositol 1,4,5-trisphosphate (IP3). IP3 interacts with its receptors in the ER membrane and induces ER Ca^2+^ release. The subsequent transient decrease in ER Ca^2+^ content is sensed by STIM1, which oligomerizes and activates SOCs, thereby inducing a Ca^2+^ influx from the extracellular compartment (Prakriya and Lewis, 2015; Albarran et al., 2016). This Ca^2+^ influx through SOCs, called store-operated Ca^2+^ entry (SOCE), not only provides Ca^2+^ to refill the ER, but also, importantly, drives a wide set of biological processes by elevating the cytosolic Ca^2+^ concentration for minutes to hours. This Ca^2+^ signal enables the recruitment of cellular effectors and Ca^2+^-dependent signaling cascades.

Due to their capacity to trigger cell signaling, SOCs perform pleiotropic functions in various cell types such as endothelial cell proliferation (Dragoni et al., 2011), lymphocyte proliferation (Vaeth et al., 2017), keratinocyte differentiation (Darbellay et al., 2009; Vandenberghe et al., 2013), mesenchymal stem cell migration (Peng et al., 2016) or skeletal muscle development (Louis et al., 2008). Loss of Orai1 in knockout mice and in Orai1-deficient patients leads to severe immune defects along with impairments in skeletal muscle development and alterations in hair cell function or skin integrity (Feske et al., 2006; Gwack et al., 2008; Feske, 2010). TRPC1 also plays major roles in tissue homeostasis as its genetic invalidation in mice leads to major defects of regeneration in the muscle or intestinal mucosa (Rao et al., 2010; Zanou et al., 2012). In the brain, pharmacological blockade and genetic invalidation of SOCs decrease neural stem/progenitor cell proliferation in the subventricular zone (Somasundaram et al., 2014; Domenichini et al., 2018) and impair hippocampal precursor cell proliferation (Li et al., 2012). Because NSCs in the different neurogenic brain areas can display specific responses to extracellular signals (Garofalo and Surmacz, 2006; Garza et al., 2008; Segura et al., 2015), we undertook this study to determine whether SOCEs may represent a common regulatory mechanism in adult brain NSCs. To this aim, we analyzed the presence and role of SOC in area postrema NSCs. Furthermore, because the area postrema is a critical center for energy expenditure control in response to leptin (Hayes et al., 2010), and because leptin can regulate intracellular Ca^2+^ levels (Seufert et al., 1999; Fortuño et al., 2002; Shanley et al., 2002; Kohno et al., 2007, 2008; Qiu et al., 2010; Dhar et al., 2014), we determined the impact of leptin on Ca^2+^ entry through SOCs into NSCs in the area postrema.

## 2. Materials and methods

### 2.1. Animals

Experiments were performed on adult (3–6 months old) C57BL/6J mice (Janvier Laboratories, France). Animals were housed in animal care facilities (PREBIOS, University of Poitiers; UEPAO, Centre INRAe Val-de-Loire). Experimental procedures were carried out in accordance with the guidelines of the French Ministry of Agriculture and the European Communities Council Directive and validated by the Regional Ethical Committee (Authorization no. 02184.01).

### 2.2. Solutions and chemicals

SKF-96365 and YM-58483 were purchased from Sigma Aldrich (Saint-Louis, MO, USA) and dissolved in water and DMSO, respectively. GSK-7975A from Aobious (Gloucester, MA, USA) was dissolved in DMSO. Leptin was purchased from R&D Systems (Minneapolis, MN, USA) and dissolved in water. Cell culture media and growth factors were from Invitrogen (Carlsbad, CA, USA).

### 2.3. Area postrema NSC cultures

**FIGURE 1:**
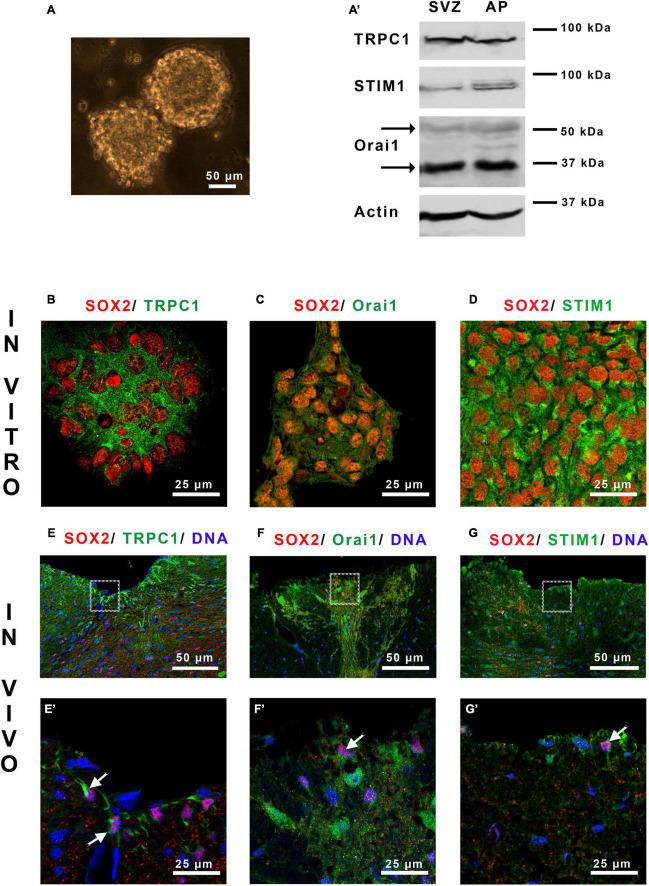
Area postrema NSCs express SOCs. **(A)** Micrograph of area postrema neurospheres and Western blot analysis **(A’)** of TRPC1, Orai1 and STIM1 proteins obtained from neurospheres of the subventricular zone (SVZ), used as a positive control, and of area postrema (AP). The molecular weights of the markers are given on the right side of the figure. The arrows point to the two bands obtained for Orai1 that correspond to unglycosylated (35 kDa) and glycosylated (50 kDa) forms of this protein. **(B–G’)** Micrographs representing immunostainings of SOX2 (red) with TRPC1 [green, **(B,E,E’)**], Orai1 [green, **(C,F,F’)**], or STIM1 [green, **(D,G,G’)**] in area postrema NSC cultures **(B–D)** or on mouse brain sections **(E–G,E’–G’)**. Cell nuclei were labeled with DAPI (blue). **(E’–G’)** Correspond to higher magnifications of the area postrema outlined in **(E–G)**, respectively. In **(E’–G’)** arrows point to doubly stained cells.

Area postrema was microdissected out of C57BL/6J adult mice brains, incubated in accutase for 15 min at 37°C and then mechanically dissociated as single cells that were grown in DMEM-F12 supplemented with 1% B27 and with 20 ng.ml^–1^ EGF and 10 ng.ml^–1^ FGF2. Neurospheres were grown in suspension and obtained after 1 week *in vitro* (Figure 1A). For replating, neurospheres were collected, mechanically dissociated as single cells and seeded to obtain secondary neurospheres that were used for Ca^2+^ imaging, RT-PCR, western blotting, immunostaining and cell proliferation assays.

### 2.4. Western-blot

For western blotting analysis, area postrema neurospheres were lysed in Laemmli loading buffer (Sigma Aldrich) and heat denatured at 95°C for 5 min, in parallel with subventricular zone neurospheres used as positive controls (Domenichini et al., 2018). All samples were resolved in a 9% sodium dodecyl sulfate-polyacrylamide gel along with pre-stained standard molecular weight markers (BioRad Laboratories, Hercules, CA, USA). Proteins were transferred to nitrocellulose membranes (0.20 μm-pore size; GE Healthcare, Little Chalfont, UK), using a mini protean electroblotter (BioRad Laboratories). After three washes in TBS (20 mmol.l^–1^ Tris–HCl, 150 mmol.l^–1^ NaCl, pH 7.5) containing 0.1% (volume/volume) Tween 20 (TBS-Tween), immunoblots were probed overnight at 4°C in TBS-Tween with 3% (weight/volume) fat milk with either rabbit polyclonal anti-Orai1 (H-46, Santa Cruz Biotechnology, Dallas, TX, USA; 0.4 μg.ml^–1^), or mouse monoclonal anti-TRPC1 (E-6, Santa Cruz Biotechnology; 0.4 μg.ml^–1^), or mouse monoclonal anti-STIM1 (GOK 610954, BD Biosciences, Franklin Lakes, NJ, USA; 0.4 μg.ml^–1^), or rabbit polyclonal anti-actin (A2066, Sigma Aldrich; 1/5000). Membranes were then incubated for 1 h at 4°C with anti-mouse or anti-rabbit immunoglobulin-HRP-linked (NA931V and NA934V, respectively, 1/5000; GE Healthcare) before detection of the bound antibodies using the ECL enhanced chemiluminescence system (Immobilon, Millipore, Billerica, MA, USA). Results were analyzed with GeneGnome XRQ (SYNGENE Ozyme, Cambridge, UK).

### 2.5. Immunostaining

Immunostaining was performed on the one hand, on area postrema neurospheres thrown on glass slides with a cytospin and fixed in methanol at −20°C, and on the other hand, on adult mouse brain sections. For brain sections, mice were deeply anaesthetized and transcardially perfused with 0.9% NaCl solution followed by phosphate-buffered 4% paraformaldehyde solution at 4°C during 10 min. Brains were removed, post-fixed for 1 h at 4°C, and cryoprotected in phosphate-buffered 30% sucrose solution at 4°C overnight before cutting 20 μm-thick sections using a cryostat (Leica 2500).

For immunostaining, preparations were permeabilized and non-specific binding sites were blocked prior to incubation with a goat polyclonal anti-SOX2 (Y-17, Santa Cruz Biotechnology; 2 μg.ml^–1^) or a mouse monoclonal anti-SOX2 (A-5, Santa Cruz Biotechnology; 2 μg.ml^–1^) antibody along with one of the following antibodies: rabbit polyclonal anti-Orai1 (H-46, Santa Cruz Biotechnology; 2 μg.ml^–1^), or mouse monoclonal anti-TRPC1 (E-6, Santa Cruz Biotechnology; 2 μg.ml^–1^), or mouse monoclonal anti-STIM1 (GOK 610954, BD Biosciences; 2 μg.ml^–1^). Preparations were then revealed with the appropriate Alexa fluor 555 or Alexa fluor 488 conjugated antibodies (1/200, A21432, A31570, A21206, A21202; Invitrogen). DAPI was used to label nuclei. Preparations were then analyzed with an FV-1000 spectral confocal station installed on an inverted microscope IX-81 (Olympus). The emitted fluorescence was detected by spectral detection channels between 425–475 nm, 500–530 nm, and 550–625 nm, for UV, green and red fluorescence, respectively.

### 2.6. Intracellular Ca^2+^ measurements

**FIGURE 2:**
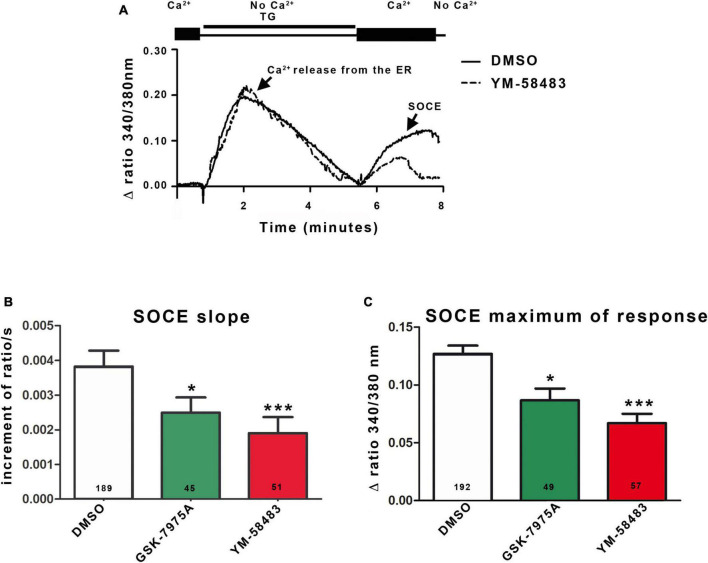
Area postrema NSCs display SOCEs. **(A)** Representative trace of SOCEs recording in area postrema NSCs. Cells were loaded with Fura-2AM and transferred to a Ca^2+^-free solution containing 4 μmol.l^–1^ thapsigargin (TG). Passive Ca^2+^ leakage from the ER and blockade of Ca^2+^ uptake to the ER by thapsigargin lead to the Ca^2+^ release from the ER corresponding to the first peak of the curve. Replacement of the zero Ca^2+^ solution with 1.8 mmol.l^–1^ Ca^2+^ physiological buffer induces Ca^2+^ entry through the SOC corresponding to the second peak (SOCE). When the cells were then placed in zero Ca^2+^ solution, the fluorescent signal returned to the initial level. Graphs representing the mean initial slope of the rising phase of the second peak **(B)** or the mean maximum of the second peak **(C)**. SOCE were recorded in 1.8 mmol.l^–1^ Ca^2+^ solution alone (control) or supplemented with YM-58483 (1 μmol.l^–1^), GSK-7975A (5 μmol.l^–1^), or DMSO (control). In **(B,C)**, the number of cells recorded in each condition is indicated in the bars of the plots. Data are expressed as mean ± s.e.m. *p < 0.05, ***p < 0.001.

Neurospheres were dissociated and 30,000 cells were plated as single cells at on fibronectin-coated glass coverslips. After 2 h, the cells were incubated for 30 min at 37°C with 3 μmol.l^–1^ of the Ca^2+^ sensitive probe Fura-2AM (Santa Cruz Biotechnology). Assays were performed in standard external buffer solution (130 mmol.l^–1^ NaCl, 5.4 mmol.l^–1^ KCl, 0.8 mmol.l^–1^ MgCl_2_, 10 mmol.l^–1^ HEPES, 5.6 mmol.l^–1^ D-glucose, pH 7.4) supplemented with 1.8 mmol.l^–1^ Ca^2+^ or 0.1 mmol.l^–1^ EGTA for the Ca^2+^- free solution. Cells were perfused with the buffer solution containing 0.1 mmol.l^–1^ EGTA (Ca^2+^-free solution) and SOCE was triggered with thapsigargin (Sigma Aldrich, 4 μmol.l^–1^). For experiments with leptin, area postrema cells were incubated with 6.25 nmol.l^–1^ leptin during 30 min at 37°C prior to intracellular Ca^2+^ measurements. Fluorescence images were recorded using a lambda 421 coupled to an Olympus IX73 inverted microscope and a Zyla sCMOS 4.2 PLUS camera. Consecutive excitation of Fura-2AM at 340 nm and 380 nm was undertaken every 2 s, and emission fluorescence was collected at 505 nm at 37°C using the Metafluor software. The 340/380 nm fluorescence ratio was measured over time in selected regions of interest (ROI) on several cells whose numbers are given in Figures 2B, C, 4B. The fluorescence ratio (340/380 nm) was measured in each cell and subtracted from the value obtained after store depletion, just at the time of Ca^2+^ re-addition (This value is expressed as Δ ratio 340/380 nm on the figures).

### 2.7. Cell proliferation assays

**FIGURE 3:**
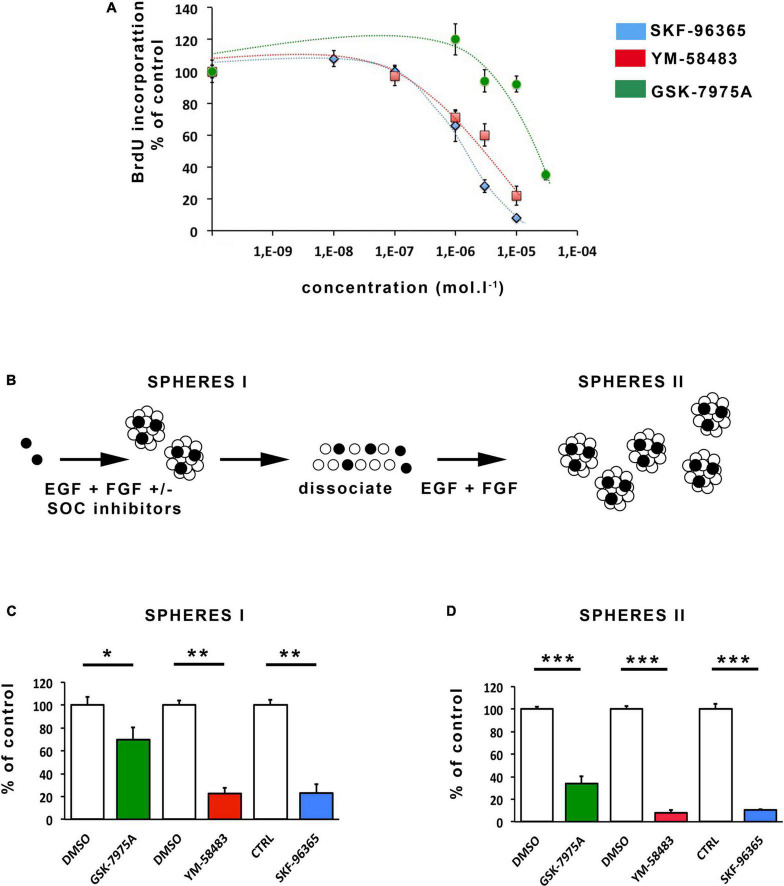
Pharmacological inhibition of SOCs inhibits proliferation and self-renewal of area postrema NSCs. **(A)** Percentages of BrdU incorporation into cells maintained for 24 h in the presence of increasing concentrations of SKF-96365 (in blue), YM-58483 (in red) or GSK-7975A (in green). Data represent means ± s.e.m of 4 experiments, each condition being assessed in quadruplicates within each experiment. **(B)** Scheme of the assay used for analysis of NSC activation and self-renewal. Data obtained with this assay are represented in **(C,D)**. Stem cells are represented by black-colored circles and progenitors by white-colored circles. Number of primary neurospheres [**(C)**, SPHERES I] obtained following 1 week of culture with 20 μmol.l^–1^ GSK-7975A (in green), 5 μmol.l^–1^ YM-58483 (in red), 1 μmol.l^–1^ SKF-96365 (in blue), or DMSO (used as control for GSK-7975A and for YM-58483). Number of secondary neurospheres [**(D)**, SPHERES II] obtained following replating. Data are expressed as percentage of control and represent means ± s.e.m of at least 2 experiments, each condition being assessed in triplicate within each experiment. *p < 0.05, **p < 0.01, ***p < 0.001.

Cell proliferation was determined by a bromodeoxyuridine (BrdU) incorporation assay. To this aim, area postrema neurospheres were dissociated as single cells and plated at the concentration of 30,000 cells.ml^–1^ in poly-L-Lysine-coated 96 well plates. The cells were treated for 24 h with SKF-96365, YM-58483 or GSK-7975A used at the concentrations ranging, respectively, from 10 nmol.l^–1^ to 10 μmol.l^–1^, 100 nmol.l^–1^ to 10 μmol.l^–1^ or 1 μmol.l^–1^ to 30 μmol.l^–1^, as indicated in Figure 3A. BrdU was added to the medium for the last 4 h of the assay. BrdU incorporated into DNA was quantified according to the manufacturer’s instructions (Roche Diagnostics, Meylan, France).

### 2.8. Neurosphere formation and self-renewal assays

The area postrema was dissected from mouse brains, dissociated, and seeded at 10,000 cells per ml in 24-well plates in cell culture medium supplemented with any of the following treatments: SKF-96365 (1 μmol.l^–1^), YM-58483 (5 μmol.l^–1^), GSK-7975A (20 μmol.l^–1^) or leptin (6.25 nmol.l^–1^). Controls were performed with culture medium alone (for SKF-96365 and for leptin) or supplemented with DMSO (solvent of YM-58483 and GSK-7975A that was diluted like the drugs i.e., 1/10,000 for YM-58483 and 1/2,500 for GSK-7975A). Primary neurospheres were allowed to grow during 1 week and then counted under a microscope. For self-renewal assays, area postrema primary neurospheres were collected, dissociated as single cells, and reseeded in culture medium to obtain secondary neurospheres.

### 2.9. Data analysis

Statistical significance of differences was examined by one-way analysis of variance (ANOVA) followed by the Bonferroni’s *post-hoc* test for multiple comparisons, or by non-parametric Mann and Whitney test for pairwise comparisons (Statview 5.00 software). The statistical significance level was set for *p*-values < 0.05 and represented on the figures by: *p < 0.05, **p < 0.01, ***p < 0.001.

## 3. Results

### 3.1. Area postrema NSCs display SOCEs

Mainly consisting of Orai1, SOCs are activated by the Ca^2+^ sensor STIM1 at the ER membrane and can mobilize also TRPC1 which will amplify the Ca^2+^ entry. Western blot (Figure 1A’) analysis shows that neurospheres from area postrema express TRPC1, Orai1 and STIM1 (Figure 1A). These results were confirmed by immunostainings (Figures 1B–D), which furthermore highlights the presence of SOC molecular actors in neural stem-like cells expressing SOX2. The physiological relevance of these data obtained *in vitro* was then investigated on mice brain sections. Figures 1E–G and their corresponding magnifications in Figures 1E’–G’ illustrate that TRPC1, Orai1 and STIM1 are found in area postrema cells *in vivo* within SOX2-expressing stem cells.

Next, we assessed the functionality of SOCs in area postrema NSCs by determining their ability to support Ca^2+^ entries in response to depletion of ER Ca^2+^ stores. To this end, cells were loaded with Fura-2AM, a Ca^2+^ sensitive ratiometric dye that binds to free intracellular Ca^2+^. To deplete ER Ca^2+^ stores, we used thapsigargin that blocks the cell’s ability to pump Ca^2+^ into the ER through the sarco-endoplasmic reticulum calcium-ATPase (SERCA). Figure 2A (first peak) shows that exposure to thapsigargin (TG) in a zero Ca^2+^ solution induces an increase in intracellular Ca^2+^ concentration in area postrema NSCs due to inhibition of Ca^2+^ uptake and a passive leak of Ca^2+^ from the ER. The ER store-depletion is sensed by STIM1 and secondarily activates SOCs at the plasma membrane. Although SOCs open within a few tenths of a second after Ca^2+^ release from the endoplasmic reticulum, no Ca^2+^ can enter the cell as long as the cell is maintained in a Ca^2+^-free buffer solution. After Ca^2+^ release from ER was over, Ca^2+^ was added to the extracellular medium and permitted a Ca^2+^ influx into the cell (SOCE, Figure 2A, second peak). To quantify the SOCEs, the initial slopes observed just after addition of Ca^2+^ were measured on traces obtained from multiple cells. In addition, we quantified the maximum value of the second peak, even though this parameter depends both on Ca^2+^ entries through SOCs, on processes that extrude Ca^2+^ from cells and on uptake in the mitochondria. Quantification of the slope and maximum of response showed a decrease of about 35% for GSK-7975A (5 μmol.l^–1^) and 46% for YM-58483 (also known as BTP2; 1 μmol.l^–1^) compared to control (DMSO). These results indicate that SOC inhibitors decreased SOCE, confirming the presence of functional SOCs in area postrema cells (Figures 2B, C).

Taken together, our data establish that area postrema NSCs are endowed with functional SOCs.

### 3.2. SOCEs control the proliferation and self-renewal of area postrema NSCs

As SOCs are known to influence neurogenesis in the subventricular zone, we investigated whether they could regulate NSC proliferation and self-renewal in area postrema by determining the impact of their pharmacological blockade. Cell proliferation was assessed by measuring BrdU incorporation into DNA during the S phase of the cell cycle. For this assay, the SOC inhibitors, YM-58483, GSK-7975A, and SKF-96365 were used (Li et al., 2012; Aulestia et al., 2018; Domenichini et al., 2018; Terrié et al., 2021). The results shown on Figure 3A demonstrate that SKF-96365, YM-58483 and GSK-7975A, dose-dependently decrease area postrema cell proliferation for concentrations ranging from 0.1 to 10 μmol.l^–1^, and 0.1 to 10 μmol.l^–1^, and 3 to 30 μmol.l^–1^, respectively, which is within the range of concentrations required for these inhibitors to induce cellular effects (Prakriya and Lewis, 2015).

Neural stem cells are characterized *in vitro* by their capacity to form neurospheres and self-renew when maintained in the presence of mitogens. To investigate the possible regulatory role of SOCs on the activation and self-renewal of area postrema NSCs, freshly dissociated area postrema cells were plated at the concentration of 10,000 cells per ml in the absence (control) or presence of the SOC inhibitors SKF-96365, YM-58483, and GSK-7975A (Figure 3B) used at concentrations of 1, 5, and 20 μmol.l^–1^, respectively, which still allow mitotic activity (Figure 3A). Neurospheres were allowed to develop for 1 week and then counted. The number of neurospheres (primary neurospheres indicated as SPHERE I in Figure 3B) obtained reflects the activation of NSCs in the culture. Primary spheres encompass progenitors (represented in white on Figure 3B) resulting from stem cell proliferation, and stem cells (depicted in black in Figure 3B) resulting from self-renewal, i.e., proliferation and maintenance of a stem cell phenotype. To quantify stem cells in primary neurospheres and thus analyze the self-renewal process, we determined the number of new neurospheres at the next generation (Azari et al., 2011). To this aim, primary neurospheres obtained in each experimental condition were, respectively, collected, dissociated, plated and allowed to develop into secondary neurospheres (Figure 3B, SPHERE II) in the culture medium without addition of SOC inhibitors. Our results show that the number of primary neurospheres (Figure 3C) and secondary neurospheres (Figure 3D) are dramatically reduced by the addition of SKF-96365, YM-58483, and GSK-7975A, indicating that SOCs play a major role in both mitotic stimulation and self-renewal of NSCs in the area postrema.

### 3.3. Leptin reduces SOCEs and impairs NSC self-renewal

Store-operated Ca^2+^ entries are evoked and modulated by various extracellular signals, including hormones (Dragoni et al., 2011; Rodríguez-Moyano et al., 2013; Somasundaram et al., 2014; Zuccolo et al., 2018; Núñez et al., 2019; Lv et al., 2020). Among them, leptin is an adipose tissue-derived hormone that controls food intake and energy expenditure by acting on peripheral organs and brain structures (Morton et al., 2014). The area postrema plays a major role in leptin control of energy homeostasis (Hayes et al., 2010). Leptin used at physiological nanomolar concentrations has been shown to reduce Ca^2+^ influx in several cell types, including neurons (Seufert et al., 1999; Fortuño et al., 2002; Shanley et al., 2002; Kohno et al., 2007, 2008; Sun et al., 2019).

**FIGURE 4:**
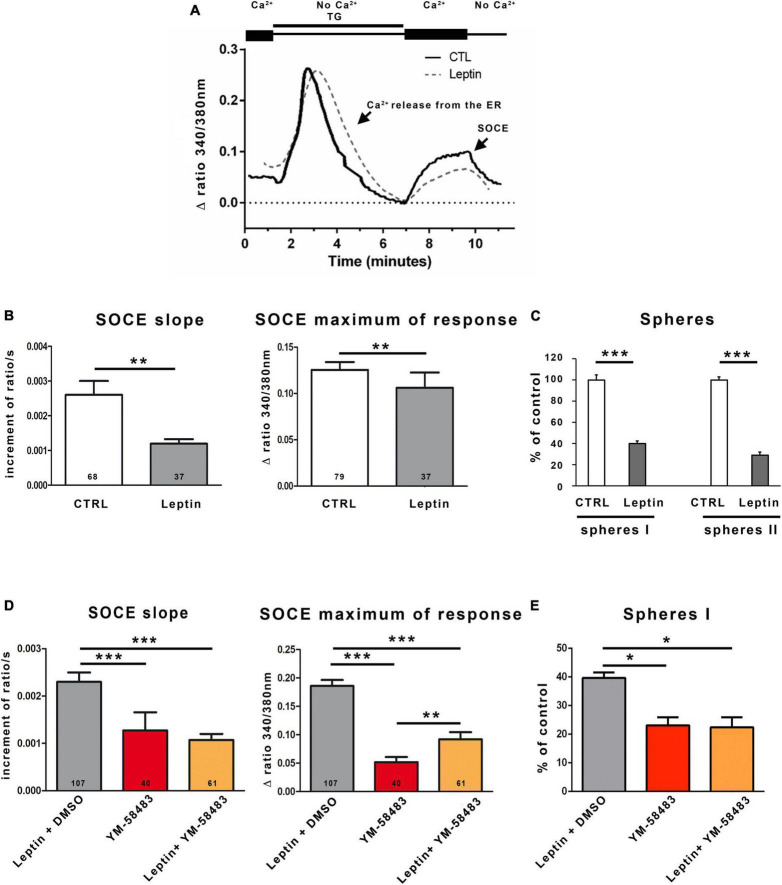
Leptin reduces SOCEs and self-renewal of area stem postrema NSCs. **(A)** Representative trace of SOCEs recorded in area postrema NSCs in the absence (solid line) or following a pre-incubation with 6.25 nmol.l^–1^ leptin (dashed line). **(B)** Graphs representing the average initial slope of the ascending phase during the second peak or the average maximum of the second peak recorded in the absence (CTRL) or presence of leptin (Leptin). The number of cells recorded in each condition is indicated within the bars. Data are represented as means ± s.e.m. **p < 0.01. **(C)** Area postrema cells were subjected to the neurosphere assay that was performed in the absence (CTRL) or presence of 6.25 nmol.l^–1^ leptin (Leptin). Number of primary neurospheres (spheres I) obtained following 1 week of culture in the absence (CTRL) or presence of 6.25 nmol.l^–1^ leptin (Leptin), and secondary neurospheres (spheres II) obtained following replating. Data are expressed as percentage of control and represent means ± s.e.m of 2 experiments, each condition being assessed in triplicate within each experiment ***p < 0.001. **(D)** Graphs representing the average initial slope of the ascending phase during the second peak or the average maximum of the second peak recorded in the presence of 6.25 nmol.l^–1^ leptin and DMSO (Leptin + DMSO, in gray; DMSO is the solvent of YM-58483), 5 μmol.l^–1^ YM-58483 (YM-58483, in red), or 6.25 nmol.l^–1^ leptin and 5 μmol.l^–1^ YM-58483 (Leptin + YM-58483, in orange). The number of cells recorded in each condition is indicated in the bars of the plots. Data are represented as means ± s.e.m. **p < 0.01, ***p < 0.001. **(E)** Area postrema cells were subjected to the neurosphere assay performed in the presence of 6.25 nmol.l^–1^ leptin and DMSO (Leptin + DMSO, in gray), 5 μmol.l^–1^ YM-58483 (YM-58483, in red), or 6.25 nmol.l^–1^ leptin and 5 μmol.l^–1^ YM-58483 (Leptin + YM-58483, in orange). Number of primary neurospheres (spheres I) obtained following 1 week of culture. Data are expressed as percentage of control and represent means ± s.e.m of 2 experiments. *p < 0.05.

As we observed that area postrema NSCs express the leptin receptor (Supplementary Figure 1), a possible effect of leptin on SOCs was investigated. SOCEs were measured in cells preincubated with 6.25 nmol.l^–1^ leptin during 30 min before Ca^2+^ imaging. Figure 4A depicts a Ca^2+^ trace obtained in the absence (full line) or presence (dashed line) of leptin. Quantification of the initial slope and maximum of the traces reveals that leptin significantly reduces SOCEs of 54 and 15%, respectively, compared to control (Figure 4B).

Consistent with the effects of leptin on SOCEs (Figures 4A, B) and the data of Figures 3C, D showing that an inhibition of SOCs results in decreased activation of area postrema NSCs, leptin was found to reduce both sphere formation and self-renewal of area postrema NSCs (Figure 4C). Furthermore, co-treatment of area postrema NSCs with leptin and the SOC inhibitor YM-58483 amplified neither YM-58483 effects SOCEs (that was decreased by about 50% for the slope in both YM-58483 and YM-54843 + leptin and between 70 and 50% for the maximum of response for YM-58483 and YM-54843 + leptin, respectively, compared to control) (Figure 4D) nor its effects on neurospheres (Figure 4E), suggesting leptin exerts its effects on area postrema NSCs by inhibiting SOCs. Regarding the maximal response, it was unexpectedly slightly higher when YM-58483 was combined with leptin than when YM-58483 was used alone, which may be related to the fact that the maximal response depends on Ca^2+^ entries through SOCs and could secondarily modulate other Ca^2+^ processes.

## 4. Discussion

By combining multiple approaches, our study establishes for the first time, to the best of our knowledge, that area postrema NSCs express SOCs that support Ca^2+^ entries involved in NSC activation and self-renewal. In addition, our data provide evidence that leptin, an adipocyte-derived hormone whose effects on energy homeostasis depend on area postrema (Morton et al., 2014), inhibits SOCEs and impairs self-renewal of area postrema NSCs. Along with studies performed on the adult subventricular zone and hippocampus, which are the two major NSC niches in adult mammalian brain (Li et al., 2012; Somasundaram et al., 2014; Garcia-Alvarez et al., 2015; Domenichini et al., 2018; Coronas et al., 2020), our results obtained on the area postrema emphasize that SOCs represent major regulators of NSC activity in different neurogenic niches of the adult brain.

In the present study, we detected TRPC1, Orai1 and STIM1 expression in cells stained with the stem cell marker SOX2, both in area postrema neurosphere cultures and *in vivo* in brainstem sections, supporting that area postrema NSCs are endowed with proteins than can form SOCs and with their activator STIM1. In addition to STIM1, STIM2 is expressed in the brain, particularly in the hippocampus and cortex and may contribute to SOCE (Kraft, 2015; Moccia et al., 2015). However, STIM2 is mostly expressed in mature neurons while STIM1 predominates in immature cells (Kushnireva et al., 2020). In our study, we observed STIM1 expression in area postrema NSCs, which is consistent with the previously described essential role of STIM1 in brain neural stem cells, as STIM1 knockdown leads to loss of SOCE, decreased proliferation and early spontaneous differentiation of these immature cells (Somasundaram et al., 2014; Gopurappilly et al., 2018). Area postrema cells responded to Ca^2+^ stores depletion by inducing Ca^2+^ entry that was blocked by SOC inhibitors, showing that SOCs in area postrema NSCs are functional. Using pharmacological inhibitors of SOCs, we established that SOCs are required for cell proliferation and self-renewal in area postrema NSCs, as they are for NSCs of other major neurogenic niches, namely the subventricular zone and hippocampus (Li et al., 2012; Somasundaram et al., 2014; Domenichini et al., 2018), suggesting that this Ca^2+^-related mechanism is conserved across different neurogenic niches in the adult brain to maintain NSC pools. Recent data have indeed unveiled a central role of Ca^2+^ in stemness control (Luchsinger et al., 2019; MacDougall et al., 2019). Specifically, high intracellular levels of Ca^2+^ have been reported to maintain pluripotency in embryonic stem cells whereas low intracellular Ca^2+^ improves the hematopoietic stem cell maintenance (Luchsinger et al., 2019; MacDougall et al., 2019). Although the involvement of Ca^2+^ in the maintenance of stemness may depend on the stem cell studied, possibly due to the specific characteristics of different stem cell types or the different microenvironment in which stem cells reside (Snoeck, 2020), our study as well as data from the literature emphasize the central role played by Ca^2+^ signals in stem cells. In this context, SOCs that can be recruited by numerous extracellular signals (Prakriya and Lewis, 2015) may represent a pivotal mechanism for transducing and integrating various extracellular inputs to adapt NSC behavior to physiological conditions.

Area postrema is a circumventricular organ lacking a blood–brain barrier and is a critical homeostatic integration center for humoral signals to the brain. Specifically, the area postrema controls energy expenditure in response to the adipocyte-derived hormone leptin (Hayes et al., 2010). Our data provide evidence that, in addition to its previously reported effects on area postrema neuron excitability (Smith et al., 2016), leptin reduces self-renewal of area postrema NSCs. A similar effect of leptin has been described in subventricular zone NSCs with decreased stem cell capacities following NSC exposure to leptin (Segura et al., 2015). Conversely, in leptin receptor knockout mice (db/db mice) (Ramos-Rodriguez et al., 2014) or in physiological models of leptin deficiency achieved by long-term intermittent fasting (Manzanero et al., 2014; Dias et al., 2021), loss of leptin effect promoted proliferation of NSCs in the subventricular zone and hippocampus. Because SOCs display a major role in the NSC maintenance, the effect of leptin on SOCs was investigated. Our data show that leptin inhibits Ca^2+^ entry through SOCs into area postrema NSCs. Consistently, an inhibitory effect of nanomolar concentrations of leptin on Ca^2+^ entries has also been observed in cells of the pancreas (Seufert et al., 1999), hippocampus (Shanley et al., 2002), and hypothalamic arcuate nucleus (Kohno et al., 2008). Thus, the leptin-induced decrease in Ca^2+^ influx through SOCs in area postrema NSC cultures may be responsible for the leptin-triggered reduction of NSCs in area postrema. Physiologically, another metabolic hormone, amylin, has been demonstrated to exert its satiating effect via stimulation of area postrema NSC proliferation (Liberini et al., 2016a) in parallel with direct control of differentiated neurons in the same structure (Turek et al., 2010; Liberini et al., 2016b). The present findings thus point to area postrema NSC as a novel key integrator of humoral metabolic cues for appetite, food intake and body weight regulations.

Interestingly, tight links have been reported between SOCs and obesity. Obese mice display decreased SOCEs (Climent et al., 2020) while SOCs deregulation induces metabolic syndromes in obesity (Arruda et al., 2017). Given that leptin dysregulation is associated with obesity and that high-fat diet impedes neurogenesis (Park et al., 2010), our study underlines the importance of designing future studies to investigate the roles of SOCs in the context of metabolic disorders, especially obesity.

## Data availability statement

The raw data supporting the conclusions of this article will be made available by the authors, without undue reservation.

## Ethics statement

This animal study was reviewed and approved by the Regional Ethical Committee for the UEPAO, Centre INRAe Val-de-Loire (Authorization no. 02184.01).

## Author contributions

CB: collection, assembly, analysis and interpretation of data on RT-PCR, and self-renewal assays. ET and ND: collection, assembly, and interpretation on calcium imaging. PA: collection, assembly, analysis and interpretation of immunostaining data, and manuscript writing. TH: design, analysis, and interpretation of western blot data. LC: collection and analysis of cell proliferation data. BC: analysis and interpretation of calcium imaging data, and manuscript writing. EM and VC: conception and design, financial support, collection, assembly, analysis and interpretation of data, and manuscript writing. All authors approved the manuscript.
