# Supplementary material for: Neural stem cell self-renewal stimulation by store-operated calcium entries in adult mouse area postrema: influence of leptin
Source: Front Cell Neurosci. 2023 Jun 9;17:1200360. doi: 10.3389/fncel.2023.1200360 (PMC10287973; doi:10.3389/fncel.2023.1200360)
Supplement: Supplementary file 1 [file Data_Sheet_1.docx]

Supplementary Material

Neural stem cell self-renewal stimulation by store-operated calcium entries in adult mouse area postrema: influence of leptin.

**Cyrine Ben Dhaou^1*^, Elodie Terrié^2*^, Déliot Nadine^2*^, Thomas Harnois^2^, Laetitia Cousin^2^, Patricia Arnault^2^, Bruno Constantin^2^, Emmanuel Moyse^1,3#^ and Valérie Coronas^2#^**

*** Correspondence:**

Valérie Coronas
E-mail: valerie.coronas@univ-poitiers.fr

**Supplementary data**


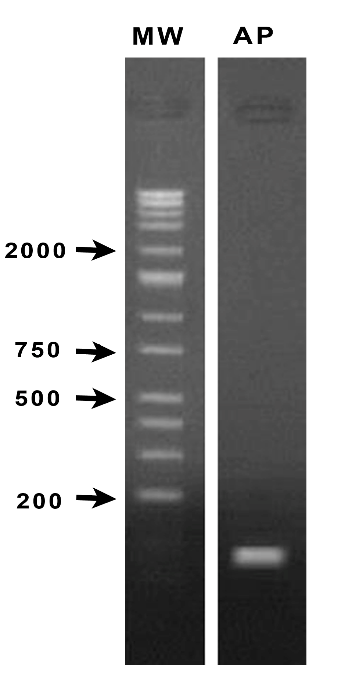


**Supplementary Figure 1:** RT-PCR detection of full-length leptin receptor (ObR) transcripts in area postrema cells (AP) cultured as neurospheres. The left lane corresponds to DNA molecular weight markers (MW); molecular weights (in bp) are indicated on the left. Total RNA was extracted and reverse-transcribed from area postrema neurospheres using a single-procedure kit for small numbers of cells: the RT SuperScript VILO^TM^ (Invitrogen 11754050, BioRad 1708897) supplemented with RNaseOUT (Invitrogen 10777-019) following manufacturer’s instructions. ObR mRNA (Leptin receptor transcript variant 2, mRNA – Mus musculus – Ref seq NM_010704) was subjected to PCR using AGAATGAAAAAGTTGTTTTGG as forward and GTGTTCATGTTCTCAAGCCTTGT as reverse primers as previously described in Tavernier et al 2014 with resulting amplicon of 121 bp (Tavernier et al. 2014). Number of PCR cycles for amplification of RT-cDNA sequences with SybBR-Green PCR were 31.82.

Supplementary reference:

Tavernier, Annabelle, Jean-Baptiste Cavin, Maude Le Gall, Robert Ducroc, Raphaël G. P. Denis, Françoise Cluzeaud, Sandra Guilmeau, et al. 2014.  Intestinal Deletion of Leptin Signaling Alters Activity of Nutrient Transporters and Delayed the Onset of Obesity in Mice . *FASEB Journal: Official Publication of the Federation of American Societies for Experimental Biology* 28 (9): 4100‑4110. https://doi.org/10.1096/fj.14-255158.
